# Sound as a stimulus in associative learning for heat stress in Arabidopsis

**DOI:** 10.1080/19420889.2020.1713426

**Published:** 2020-01-13

**Authors:** Abhishek Bhandawat, Kuldip Jayaswall, Himanshu Sharma, Joy Roy

**Affiliations:** a Agri-Biotechnology Department, National Agri-Food Biotechnology Institute, Mohali, Punjab, India; b Agriculture Biotechnology Department, ICAR-Directorate of Onion and Garlic Research, Rajgurunagar, Pune, Maharashtra, India

**Keywords:** Associative learning, conditioning, plant acoustic, plant memory

## Abstract

Plants are analogous to animals by responding physiologically and phenotypically to environmental changes. Until recently, the meaning of sound in the plant’s life remains undiscovered. In this study, we investigated the role of music in response to heat stress and its application in memory and associative learning for stress tolerance in Arabidopsis. Significant upregulation of heat-responsive genes (HSFA3, SMXL7, and ATHSP101) in response to music suggests music has an advantage during heat stress. Moreover, the defensive conditioning experiment showed that plant learns to associate music with stress (heat) and elicit better response compared to music alone. Two heat-responsive genes, HSFA3 and ATCTL1, which are well known for their interaction and regulation of an array of heat shock proteins were found to play a key role in associative learning for heat stress in Arabidopsis. Our experiment highlights the application of sound in plant conditioning and as a stress reliever. Nonetheless, the persistence of memory awaits further experiments. We foresee the potential of artificial sound as an environment-friendly stimulus in conditioning the crops for upcoming stresses and reduce the yield loss, as an alternative to breeding and genetic modifications.

## Introduction

Plants are not capable to move like animals to obtain their food or avoid unfavorable environment. In order to thrive successfully in the ever-changing environment, what they can do is learn and remember their past experiences and respond more judiciously for the future event. Evidences of learning by association are a common phenomenon observed in animals, however, in plants it has not been discovered until recently. A study conducted on pea plant revealed that plant learn to associate a neutral stimulus (wind) with blue light (food) to modulate its growth behavior [1]. The plant perceives the information (wind) advantageous as it is followed by a rewarding experience (blue light), thus it makes a decision to direct its growth in specified direction. Unlike appetitive conditioning in which plant gets a reward like blue light, defensive conditioning (in which plants develops tolerance to stress) has not been explored in plants.

Sound is a mechanical pressure wave and its significance in animal kingdom is well known [2,3]. It is just the beginning, few research groups started to investigate role of acoustics in plant kingdom [4–6]. Hypothesis-driven research has shown that plants are able to perceive external sound and produce meaningful sounds as one of the means of rapid intercellular, conspecific and even inter kingdom communications, to elicit specific response [4,6–8]. A well-established behavioral response of sound is bending of *Pisum sativum* roots toward flowing water playback sound [5]. Application of defined frequency of sound waves to improve various agronomic traits like germination rate [9], growth [10], and stress tolerance [6] has been documented. But the use of artificial sound/music for conditioning (defensive) plants to environmental stress remains elusive. Heat is one of the major constraints in crop production worldwide. It becomes essential to make the crops capable to withstand such stresses, especially when the climate change is dramatic. Heat-responsive proteins have shown to impart thermotolerance in variety of transgenic plants [11,12].

To address these questions, we performed a classical conditioning experiment in Arabidopsis wherein, we used music as a conditioned stimulus (CS) and heat stress as an unconditioned stimulus (US) and their impact on transcriptional changes in target genes. The rationale behind the experiment was to see if the plants could learn to remember and associate music with heat stress, and elicit an appropriate response measurable in terms of expressional changes in heat-responsive genes. And if this stands true, then music or other such stimulus may be exploited to devise strategies to make crops withstand harsh environmental conditions in an environment friendly manner without the need of exhaustive breeding and genetic modification strategies.

## Materials and method

### Plant material and growth conditions

*Arabidopsis thaliana* (ecotype col-0) seeds were surface sterilized with 70% ethanol for 2 minutes, then with 0.02% tween-20 for 4 minutes and washed with sterile Millipore water five times (1 minute each). The seeds were stratified at 4°C for 2d on five separate Murashige and Skoog basal agar plates, and grown under 16h day/8 h dark cycle at 22°C, light intensity 100 μmol photon m^−2^ s^−1^ in growth chamber for 12 days.

### Conditioning and sampling

**Figure 1.**
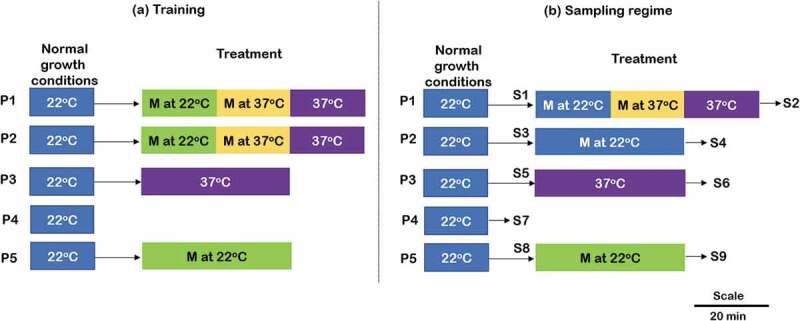
Schematic representation of conditioning/training and sampling. (a) Five days continues training (b) sampling seedlings on sixth day. P: plates containing Arabidopsis seedlings, P1: combined effect of music and heat stress, P2: effect of associative learning, P3: effect of heat stress alone, P4: Untreated control, P5: Effect of music alone. M: Music, S: sample for RNA extraction. Scale represents the duration of each treatment.

The experimental design consists of conditioning the 12d seedlings for five continuous days (twice per day: morning and evening) with specified treatments followed by sample collection on the sixth day, as indicated in Figure 1. For treatment and control experiments, three plates containing 10–15 Arabidopsis seedlings each were used (total 15 plates). Plates P1 and P2 were given green music (which comprise of classical music base and natural sound of birds, insects, and water of intensity ~ 50dB) for 20 min followed by 20 min music + heat stress followed by 20 min of heat stress alone, to study delayed conditioning response. Plate 3 (positive control) was given heat stress for 40 min to optimally induce and observe the expression of heat-responsive genes [13]. To examine the effect of music, plate 5 was given only music treatment (~ 50dB) for 40 min using wireless speakers. The duration of each treatment was kept same for all the plates. Untreated control (P4), without music and heat exposure, was also included in the study to monitor the relative expressional changes. On the sixth day, same experiment was performed as done in training except that plate 2 was given music alone to see if the plant has learned to associate music with heat stress during past 5 days conditioning/training. Three seedlings from each of the treatments (one from each plate), just prior to (S1, S3, S5, S8) and at the end of treatment period (S2, S4, S6, S9) and control (S7) were sampled for RNA extraction, as indicated in Figure 1(b).

Total RNA was extracted from seedlings using Trizol reagent (Invitrogen, USA) following the manufacturer’s instructions. The quantity and quality were assessed on Nanodrop and 1.5% Agarose gel. RNA samples with O.D_260/280_: 1.8–2.0 and intact rRNA bands on Agarose gel were selected for downstream processing. Equimolar concentration of RNA of three biological replicates was pooled before cDNA synthesis. A total of 500ng RNA was used for cDNA preparation using high fidelity first strand cDNA synthesis (Agilent Technologies, USA) with oligo-(dT)18 primers as per manufacturer’s instructions.

### Primer designing of heat-responsive genes for qPCR analysis

For evaluation of the effect of music conditioning on heat response, five heat-responsive genes, namely HSFA3 (AT5G03720.1), CHIP (AT3G07370.1), SMXL7 (AT2G29970.1), ATCTL1 (AT1G05850.1), ATHSP101 (AT1G74310.1) were downloaded from TAIR database for designing qPCR primers. The interacting partners of genes were identified using web-based STRING database (https://string-db.org/). Actin-2 (AT3G18780) was chosen as internal control. The primer pairs with Tm optimum of 60°C and product size range = 90–200bp were designed using online primer3 software (http://frodo.wi.mit.edu/). The primer details are given in Table 1.

**Table 1.** Details of primers used for qPCR analysis.

Gene name	TAIR ID	Primer sequence (5ʹ→ 3ʹ)	Tm
HSFA3	AT5G03720.1	F: GAAAAGGCGAGAAAGAAGTTCA R: CCTAAACCCTTGGTGTTCTCAG	60
CHIP	AT3G07370.1	F: TGAGGCTGCTCTTAATCAACAA R: ACAGGATCTCGGAAAATCTCAA	60
SMXL7	AT2G29970.1	F: AATGCGATTAGCGAAATTGTTT R: GATGAAGTTGTCTTGTCCACCA	60
ATCTL1	AT1G05850.1	F: TGAAAGCTGATCTCTTGAACCA R: GACAAAGATGTCGTGAGCTGAG	60
ATHSP101	AT1G74310.1	F: ACCTGATGATATTCCAGCGAGT R: AGCCAAATGAGTATCACCTCGT	60
Actin-2	AT3G18780	F: GTATTGTGCTGGATTCTGGTGA R: TGCTGTTGTGGTGAACATGTAA	60

### Realtime expression analysis of heat-responsive genes

Initially the specificity of primer pairs was inspected by semi-quantitative PCR. Single amplicon of expected size on Agarose gel suggests the specificity of primers. Realtime PCR reaction was performed with 1μl of 1:10 (v/v) diluted cDNA and Brilliant III Ultra-Fast QPCR Master Mix (Agilent Technologies, USA) in a total volume of 10μl, as per manufacturer’s instructions in CFX96 real-time thermocycler (BIO-RAD, USA). The conditions for qPCR were kept as: 95°C for 30s, 40 cycles 30 s at 95°C and 1min at 60°C. This was followed by a melt-curve program (from 60°C – 95°C) to check the specificity of the PCR amplification. The threshold cycles (C_t_) of individual target gene was normalized with C_t_ of internal control (Actin 2). No template controls were included in each run to check potential contamination of cDNA in reaction mixture components. The relative expression of genes was calculated in triplicated samples using comparative ^ΔΔ^C_t_ method [14] with respect to S7 (untreated control). The relative expression data was plotted as log2 transformed mean ± SD.

## Results and discussion

### Effect of heat on heat-responsive gene expression

The five genes included in this study are known to be involved in response to temperature stress especially heat shock. HSFA3 belongs to heat stress transcription factor family regulated by DREB2A and regulates the expression of several other heat shock proteins and is involved in establishing thermotolerance. SMXL7 has little similarity to HSP101, a ClpB chaperonin required for thermotolerance. Its transcript is free to mobilize between cells, and thus may be involved in communicating heat response between the cells. ATCTL1 encodes an endo chitinase-like protein which is crucial for tolerance to heat, salt and drought. ATHSP101 encodes a cytosolic heat shock protein involved in refolding of proteins which form aggregates under heat stress, thus has protective role against heat stress and heat stress acclimatization. It is very crucial as it interacts with various heat shock protein (HSP) family proteins, including HSP70, HSP 90, and mitochondrial. CHIP has E3 ubiquitin-protein ligase activity, interacts with ubiquitin conjugating proteins to facilitate degradation of misfolded proteins or abnormal aggregates.

**Figure 2.**
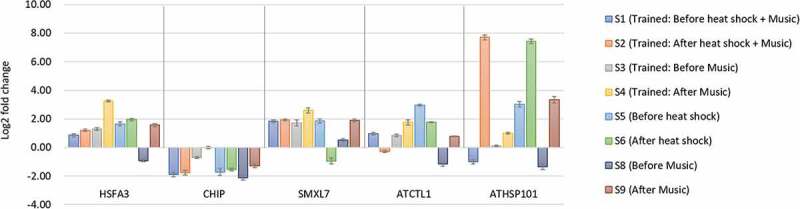
Relative expression of five heat-responsive genes in treatments (music, heat, conditioning experiments) shown as mean for triplicated experiments ± SD with respect to untreated control (S7).

Interestingly, HSFA3 and ATHSP101 genes showed increase in expression after heat stress treatment S6 (compared to S5), which suggests that plant memorize previous heat exposure and produces heightened response (Figure 2). This suggests that the, artificial sound to an extent have meaning for the plant and elicits suitable heat response at molecular level. Moreover, with respect to untreated control (S7), huge upregulation in these genes is observed both before treatment (S5) and after treatment sample (S6), and reports suggest that the transcript of HSPs usually diminishes during subsequent recovery period at ambient temperature [13]. These results suggest that plants get conditioned for heat stress (maintaining higher transcript levels of HSPs) during 5-day training period for subsequent heat stress. Moreover these two genes HSFA3 and ATHSP101, regulates heat shock response by interacting with several mitochondrial and cytosolic family of heat shock proteins in conjugation with chaperonins. These genes may be targeted for introgression to cultivated verities for a more potent heat tolerance. SMXL7, CHIP, and ATCTL1 showed lower expression in S6, while CHIP showed variable response to heat stress, which suggests that these genes have limited role in developing memory for heat stress and needs to be investigated.

### Learning by association

Arabidopsis (plate 2) were trained with music along with heat stress for 5 days and sampling on the sixth day with music alone, to see if the plant can learn to associate heat stress with music, and elicit similar heat response with music in the absence of heat stress. Amazingly, all the heat-responsive genes showed upregulation after music S4 (compared to S3), with most promising effect of HSFA3, ATHSP101, and ATCTL1, while to a lesser extent by SMXL7 and CHIP (Figure 2). Previously, association of wind with blue light to modulate growth behavior of pea plant was investigated [1]. The plant perceives the wind as important information that is followed rewarding blue light, and prefers to direct its growth in that direction. Here, we tested defensive learning in Arabidopsis for heat stress using music instead of wind. The results suggest that either plant has learned to associate stress and music or it might be the effect of music alone (discussed in later section). Nonetheless, an array of genes and pathways might be altered by sound, which invites extensive genome-wide investigation.

### Effect of music on gene expression

In order to check if the upregulation of heat-responsive genes was due to plant’s associative learning or it was just the music, we performed another pilot experiment in which only music treatment was given (plate 5; Figure 1). Surprisingly, all the heat-responsive genes showed varied degree of up-regulation after music treatment (S9) compared to (S8; Figure 2). There are reports that suggest plants perceive high intensity sounds (>80dB) and elicit specific response such as seedling growth in Arabidopsis (Johnson et al. 1998) and stress tolerance in many plants [6,10]. However, this study suggests even lower intensity sound (50 dB), is capable to impart an advantage to the plant as evident from up-regulation of heat-responsive genes. If this holds true, low-intensity sounds may be manipulated to impart advantageous effects for crop improvement without significantly affecting the natural sound perception of plants and polluting the environment.

When observed critically, S4 sample (which was trained for heat stress and music together for five continues days but given only music on the sixth day), showed higher upregulation as compared to plate given music alone (S9) especially for gene HSFA3, SMXL7, and ATCTL1. This clearly indicates that although music has played a role in upregulation, the plants that are previously trained to associate heat with music, elicits a better response. Moreover, different type of sound vibrations has shown to enhance seedling growth in mung bean, rice, cucumber and Arabidopsis [15–17]. Thus, HSFA3 and ATCTL1 appear to be promising candidates among the five tested heat-responsive genes to study defensive associative learning in Arabidopsis. No observable phenotypic change was seen in treatments with respect to control Arabidopsis seedling, which suggests plans underwent biochemical and molecular changes to minimize and prevent phenotypic changes.

### Combined effect of heat stress and music

**Figure 3.**
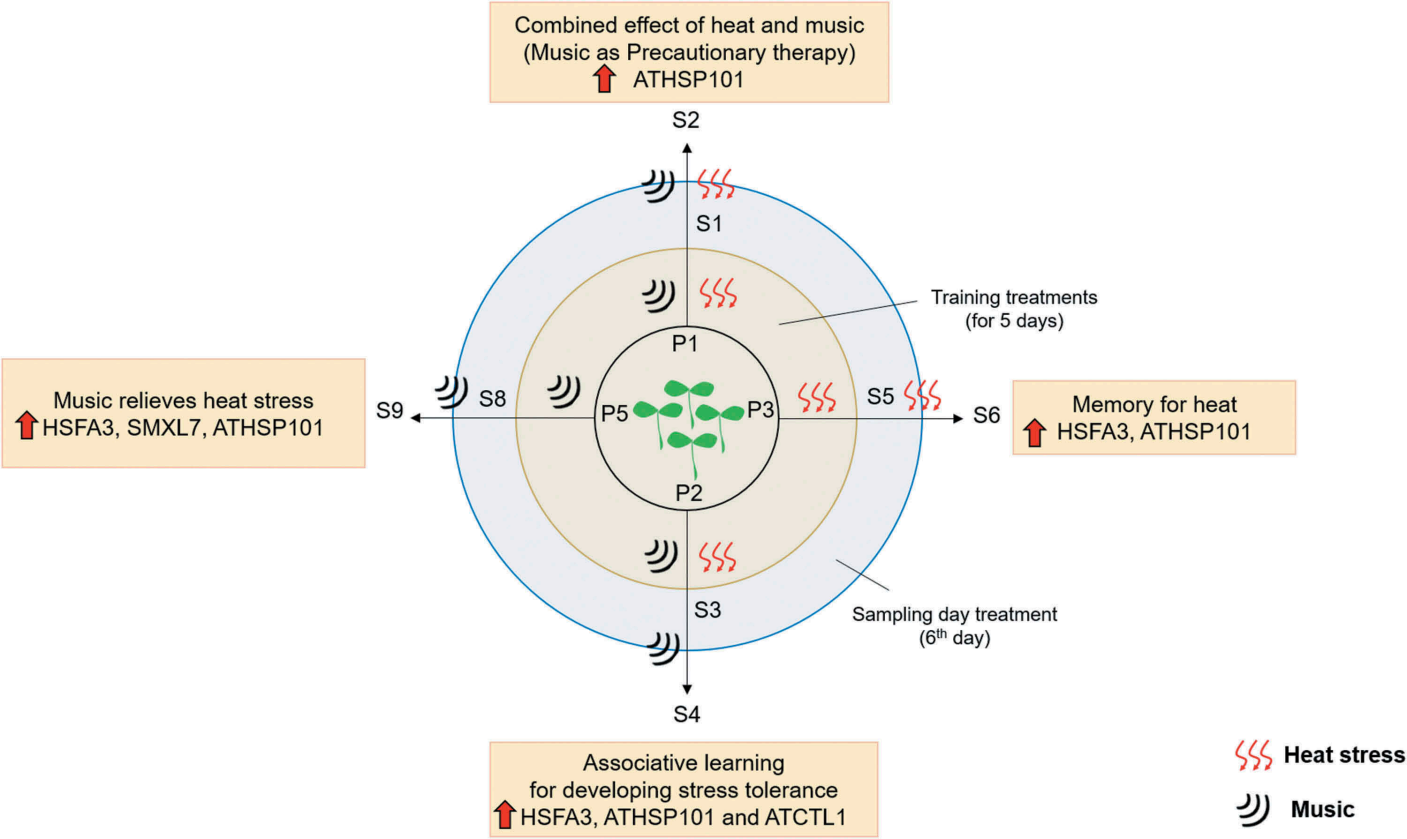
Diagrammatic summary of the effect of heat and music in heat stress memory and associative learning in Arabidopsis.

Cumulative effect of music and heat stress (plate 1) reveals that maximum upregulation is seen in S2 compared to S1 with ATHSP101. While expression of remaining genes showed little alteration. When analyzing with the samples given music alone (S9) or trained before (S4), we find that music alone or associative learning results in significant upregulation of HSPs. The promising approach to generate a better heat response and withstand heat stress is by regularly providing music as a precautionary therapy or make plants to learn to associate stress with a non-stressful stimulus like music (Figure 3). The future awaits a lot of more interesting studies in this aspect.

## Main conclusions

The preliminary research signifies the role of sound in heat-response conditioning and stress relieving in Arabidopsis. qPCR analysis reveals HSFA3 and ATCTL1 played important role in heat-music associative learning.
